# Conformational Dynamics and Stability of U-Shaped and S-Shaped Amyloid β Assemblies

**DOI:** 10.3390/ijms19020571

**Published:** 2018-02-14

**Authors:** Gianvito Grasso, Martina Rebella, Stefano Muscat, Umberto Morbiducci, Jack Tuszynski, Andrea Danani, Marco A. Deriu

**Affiliations:** 1 Istituto Dalle Molle di Studi sull’Intelligenza Artificiale (IDSIA), Scuola Universitaria Professionale della Svizzera Italiana (SUPSI), Università della Svizzera Italiana (USI), Centro Galleria 2, CH-6928 Manno, Switzerland; gianvito.grasso@supsi.ch (G.G.); andrea.danani@supsi.ch (A.D.); 2 Department of Mechanical and Aerospace Engineering, Politecnico di Torino, Corso Duca degli Abruzzi 24, IT-10128 Torino, Italy; rebella.martina@gmail.com (M.R.); s233275@studenti.polito.it (S.M.); umberto.morbiducci@polito.it (U.M.); 3 Department of Physics, University of Alberta, Edmonton, AB T6G 2R3, Canada

**Keywords:** Alzheimer’s disease, amyloid β, replica exchange, molecular dynamics, U-shaped, S-shaped, assembly, fibril, gromacs, aggregation

## Abstract

Alzheimer’s disease is the most fatal neurodegenerative disorder characterized by the aggregation and deposition of Amyloid β (Aβ) oligomers in the brain of patients. Two principal variants of Aβ exist in humans: Aβ_1–40_ and Aβ_1–42_. The former is the most abundant in the plaques, while the latter is the most toxic species and forms fibrils more rapidly. Interestingly, fibrils of Aβ_1–40_ peptides can only assume U-shaped conformations while Aβ_1–42_ can also arrange as S-shaped three-stranded chains, as recently discovered. As alterations in protein conformational arrangement correlate with cell toxicity and speed of disease progression, it is important to characterize, at molecular level, the conformational dynamics of amyloid fibrils. In this work, Replica Exchange Molecular Dynamics simulations were carried out to compare the conformational dynamics of U-shaped and S-shaped Aβ_17–42_ small fibrils. Our computational results provide support for the stability of the recently proposed S-shaped model due to the maximized interactions involving the C-terminal residues. On the other hand, the U-shaped motif is characterized by significant distortions resulting in a more disordered assembly. Outcomes of our work suggest that the molecular architecture of the protein aggregates might play a pivotal role in formation and conformational stability of the resulting fibrils.

## 1. Introduction

Proteins are complex molecular machines that undergo a huge number of conformational changes strictly related to their function. An increasing number of disorders, including Alzheimer’s (AD), Huntington’s (HD) and Parkinson’s Diseases (PD), familial British (FED) and familial Danish dementias (FDD), and type II diabetes are directly associated with the deposition of protein aggregates in tissues, including the brain, heart and spleen [1,2,3,4,5,6]. In the brain, the major components of AD-associated amyloid plaques are Aβ_1–40_ peptides but also the more toxic Aβ_1–42_ species [7], characterized by two additional amino acids and generated through a sequential cleavage of the amyloid precursor protein (APP) by β and γ secretases [8]. In general, these peptides are able to oligomerize and then the resulting oligomers can further aggregate giving rise to ordered fibrils and fibres [9]. Several experimental studies have been focused on the molecular characterization of amyloid fibrils, given the intimate relationship between molecular structure and disease onset and severity [10]. At present, all the Aβ_1–40_ species resolved by NMR, share a U-shaped motif, where the peptide chains form two β-strands connected by a loop region [11,12,13,14,15]. In case of more toxic Aβ_1–42_ species, earlier NMR models exhibited the same U-shaped motif [12]. The above mentioned molecular assembly is constituted by two β strands (involving residues V18–S26 and I31–A40) connected by a central loop region and stabilized by inter-chain H-bonds and salt bridges between residues D23–K28 [12]. More recent investigations demonstrated the possibility of S-shaped arrangements [16,17,18,19,20,21], characterized by three β strands: the N-terminal strand β1 made of residues V12–V18, the central strand β2 of residues V24–G33, and the C-terminal strand β3 of residues V36–V40. The three β strands are connected by major coil and turn regions. Interestingly, the S-shaped arrangement is not stable in case of Aβ_1–40_ species [22]. Initially, this was explained through the intra-chain salt bridge linking the side chain of K28 with the main chain of A42, which does not exist in Aβ_1–40_ [17]. Recently, it has been proposed that the cause is the lack of hydrophobic contacts in Aβ_1–40_ generated by the C-terminal residues I41 and A42 in Aβ_1–42_ peptides [23]. Within this framework, the higher toxicity of Aβ_1–42_ species compared to Aβ_1–40_ may be explained by their ability to form S-shaped assembly. Such a correlation could arise if the S-shaped model (i) was characterized by a more stable molecular architecture per se; or (ii) was able to assemble into structures that are not possible by considering the U-shaped Aβ chains, as recently suggested [22]. In this connection, a molecular level understanding of the interactions governing the structural arrangement in Aβ_1–42_ species represents an important research advance. Computational approaches such as Replica Exchange Molecular Dynamics (REMD) can be used as a powerful tool to elucidate the molecular mechanisms responsible for protein hierarchical organization. In fact, computer simulations have been widely demonstrated to be helpful in capturing mechanisms of protein folding [24,25,26] and protein-protein aggregation [27,28]. Recent computational works investigated the stability of the U-Shaped fibril models of Aβ_1–42_ and Aβ_1–40_ species [29,30]. Those studies highlighted the importance of inter-sheet side chain contacts, hydrophobic contacts among the strands and salt bridges in stabilizing U-shaped protein aggregates [29]. A further development of the above-mentioned studies might be an investigation of U-shaped and S-shaped assemblies, with the aim of comparing the structural stability and dynamics. Here, REMD was carried out to yield novel insights into the above-mentioned issue by providing a detailed conformational study of S-shaped and U-shaped Aβ_17–42_ pentamer fibril models. Several differences have been found, which clearly highlighted the S-shaped fibril as the most stable architecture due to a maximization of inter-chain hydrophobic contacts and H-bonds involving the C-terminal residues I41 and A42, in agreement with previously published reports [29]. Moreover, data concerning the U-shaped model indicated non-negligible distortions and a tendency to arrange in a more disordered fashion with respect to the S-shaped assembly.

## 2. Results

REMD simulations were carried out on the U-shaped model (Aβ_17–42_ pentamer extracted from 2BEG.pdb file [12]) and the S-shaped model (Aβ_17–42_ pentamer extracted from 2MXU.pdb file [17]) surrounded by explicitly modelled water and ions. Data analysis have been performed on the conformational ensemble at 300 K. More detailed information on simulation set up and analysis are provided in the Method Section.

### 2.1. Characterization of the Aβ Conformational Arrangements 

**Figure 1:**
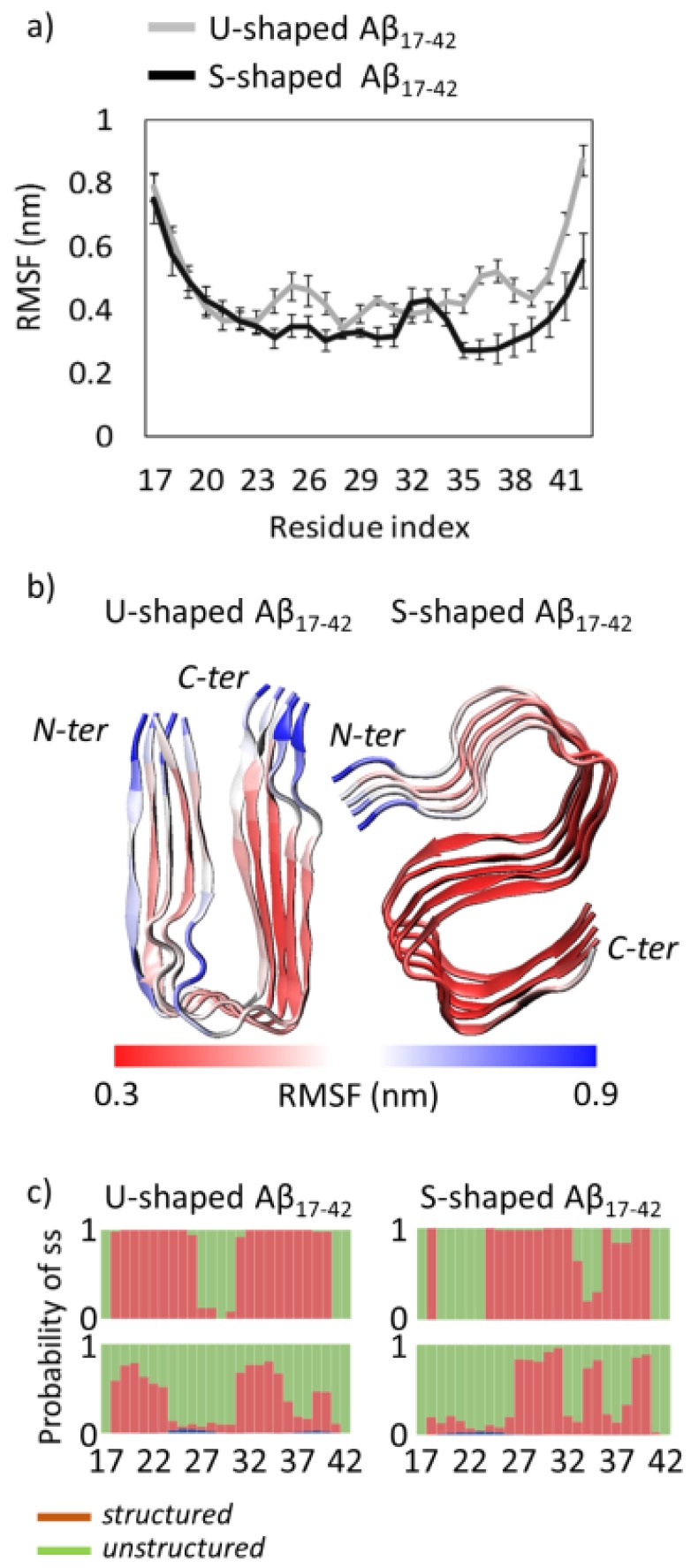
(**a**) U-shaped and S-shaped Root Mean Square Fluctuation (RMSF) of atomic positions averaged on each protein residue. Each average value and relative standard deviation was obtained by mediating the RMSF on the five considered protein chains (A–E); (**b**) U-shaped and S-shaped structural models coloured on the basis of RMSF values. The scale bar moves from red (RMSF = 0.3 nm) to blue (RMSF = 0.9 nm); (**c**) U-shaped and S-shaped residue secondary structure probability, calculated over 5 considered chains (A–E) in the PDB models (upper row) and on the Replica Exchange Molecular Dynamics (REMD) ensemble at 300 K (lower row). For the sake of clarity, the secondary structures are classified in structured (red) and unstructured (green). Moreover, the structured class does not contain helices (shown in blue) being their contribution negligible throughout the overall REMD ensemble at 300 K.

The Root Mean Square Fluctuation (RMSF) plot shows the atomic fluctuations averaged on each protein residue (Figure 1a). In both cases, as expected, terminal regions are characterized by larger fluctuations with respect to the central region due to a higher solvent exposure (Figure 1a). By comparing the two different U-shaped and S-shaped models, it is worth noticing that the main difference is located at the C-terminal tail. In particular, the protein region V36–A42 is characterized by larger fluctuations in case of the U-shaped model (RMSF_A42_ = 0.87 ± 0.10 nm), differently from what has been observed in the S-shaped model (RMSF_A42_ = 0.56 ± 0.17 nm). A visual inspection of the above-mentioned fluctuations is provided in Figure 1b. In case of the U-shaped model, peptide chains most exposed to the solvent are also characterized by higher conformational instability. Also regions V24–N27 and V36–G38 are characterized by high fluctuation peaks located on V24 (RMSF_V24_ = 0.43 ± 0.07 nm) and G37 (RMSF_G37_ = 0.52 ± 0.07 nm), respectively.

The previously highlighted conformational instability of residues V24 and G37 in the U-shaped model can be explained by analysing the secondary structure probability of the two simulated systems (Figure 1c). For each model the secondary structure has been calculated as a probability along all chains and all considered frames, as done in previous works [27]. The secondary structure probability along the REMD ensemble at 300 K (Figure 1c, lower row) was compared with the same probability in the PDB model (Figure 1c, upper row) for both U-shaped (Figure 1c, left) and S-shaped (Figure 1c, right) architectures. 

Although in both models the two predominant structures are rigid β-sheets and flexible coils, the secondary structures are differently distributed along the peptide chain. In detail, β structures are mainly located in regions V18–D23, I31–M35 and V39–V40 for the U-shaped model and N27–I31, L34–M35 and V39–V40 for the S-model. A marked loss of β-sheets was observed in both cases (U-shaped and S-shaped fibrils) if compared with the original NMR models. In detail, residues V18, V24–S26, I32, G33 and V36–G38 are characterized by a spontaneous β-coil transition in the S-shaped fibrils whereas a reduction of β-sheets was located at residues V24–S26 and V36–V40 in case of U-shaped models. It is worth mentioning that the loss of β-sheets here observed in the U-model is consistent with a previous computational study [31]. Moreover, this evidence is in line with the conformational fluctuations of residues V24 and G37 highlighted in Figure 1a. The loop domains of both U-shaped and S-shaped fibrils remain largely unstructured along the simulation trajectory, in line with the NMR starting model (Figure 1c). The only difference is located at the turn region connecting β2 (residues V24–G33) and β3 (residues V36–V40) of the S-shaped fibril. In this case, we observed an increased tendency to form a structured beta strand of residues L34–M35.

Interestingly, the total Solvent Accessible Surface Area (SASA) of the U-shaped model (76.07 ± 4.17 nm^2^) is slightly higher than that of the S-shaped model (70.62 ± 3.71 nm^2^). This result might be related to the ability of the S-shaped model to reach a more compact arrangement. The above-mentioned observation suggests that the S-model is better able to maximize intra- and inter-chain contacts. More detailed information on SASA and RG is reported in Supplementary Figure S2. 

### 2.2. Characterization of the Aβ Interatomic Interactions

**Figure 2:**
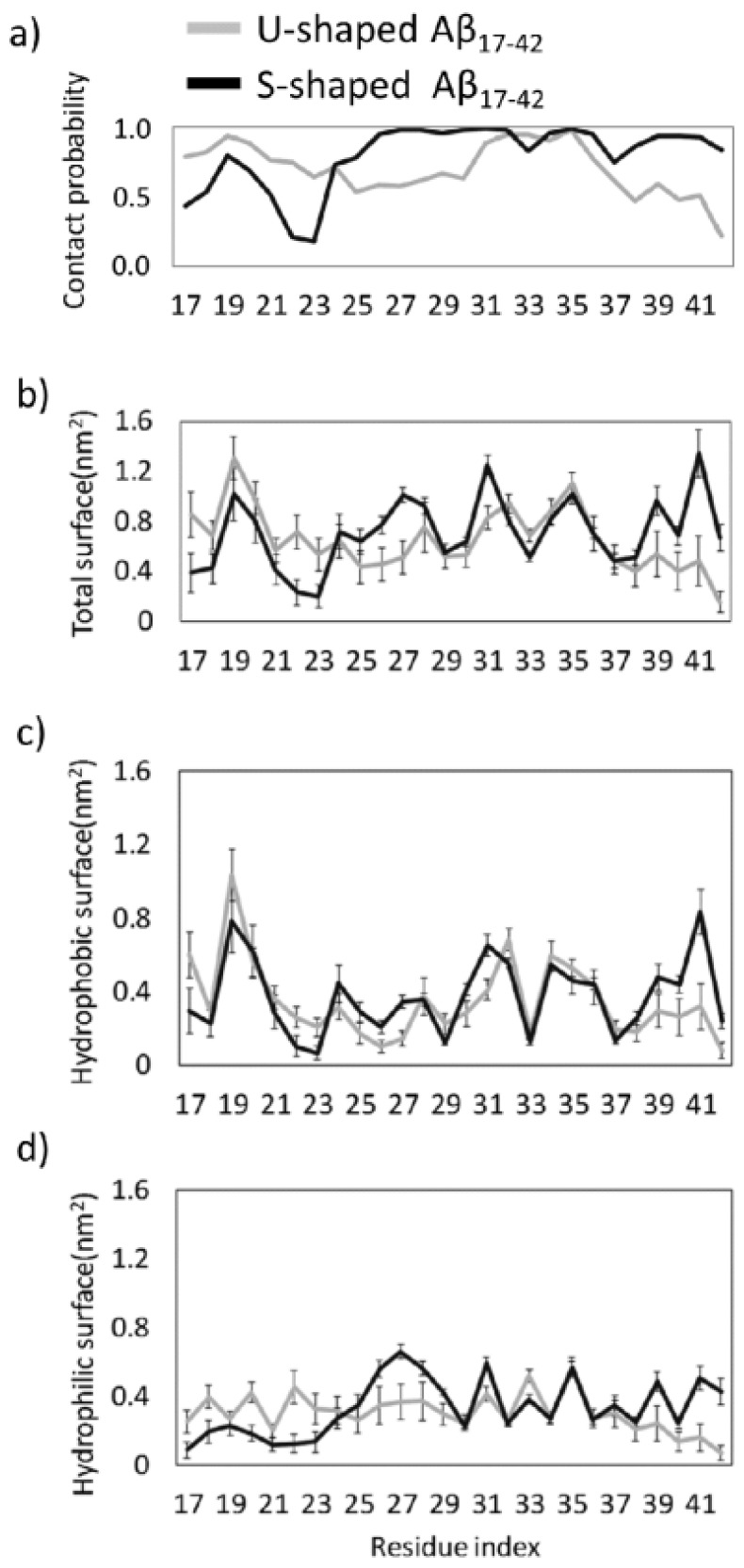
(**a**) U-shaped and S-shaped per-residue inter-chain contact probability plot; (**b**) U-shaped and S-shaped per-residue inter-chain total interacting surface; (**c**) U-shaped and S-shaped per-residue inter-chain hydrophobic interacting surface; (**d**) U-shaped and S-shaped per-residue inter-chain hydrophilic interacting surface. In all plots contacts between chains B-C and C-D were considered and averaged on the REMD ensemble at 300 K.

In order to provide a deeper understanding of the interactions leading to the above mentioned conformational properties, we have studied the detailed intra/inter-chain interatomic interactions at an atomistic level. An overall view of regions mainly involved in the inter-chain non-covalent bonds are provided by contact probability plots (Figure 2a). A lack of inter-chain interactions can be detected in different regions of both models. Regarding the S-shaped model, a slight decrease in interatomic interactions may be observed at residues L17–V18, G37 and a marked one in range A21–G25. In case of the U-shaped model, lower contact probability was observed in protein regions V24–A30 and G37–A42.

A noticeable difference between the two models is found at the C-terminal residues V39–A42, showing lower contact probability values in case of the U-shaped model (probability = 0.22) when compared with the S-shaped model (probability = 0.85). The lack of interactions indicates the presence of defects in the fibril structure. These defects, which are localized in both central and C-terminal regions in the U-shaped model may be related to a higher conformational instability with respect to the S-shaped where inter-chain contact defects are mainly localized only in region L17–D23. 

Another picture of the presence of the above mentioned local defects in inter-chain contacts is provided by a detailed analysis of the inter-chain total, hydrophobic and hydrophilic interaction surface (Figure 2b–d, respectively).

In a greater detail the U-model presented a reduced inter-chain interaction surface in both the core and C-terminal regions, whereas the S-shaped model showed a lower total surface only in the L17–D23 region. It may be of interest to decompose the total interaction surface in its hydrophobic (Figure 2c) and hydrophilic (Figure 2d) components. In the core and C-terminal region, the S-shaped model showed to maximize both hydrophobic and hydrophilic inter-chain interaction surfaces with respect to the U-shaped model, whereas the latter showed only a significantly higher hydrophilic interaction in the L17–D23 region. Hydrophilic interactions and inter-chain contacts provide an indication of hydrogen bond presence, strongly related to the conformational stability of the amyloid oligomers and fibrils as indicated by literature in this field [11,32,33].

**Figure 3:**
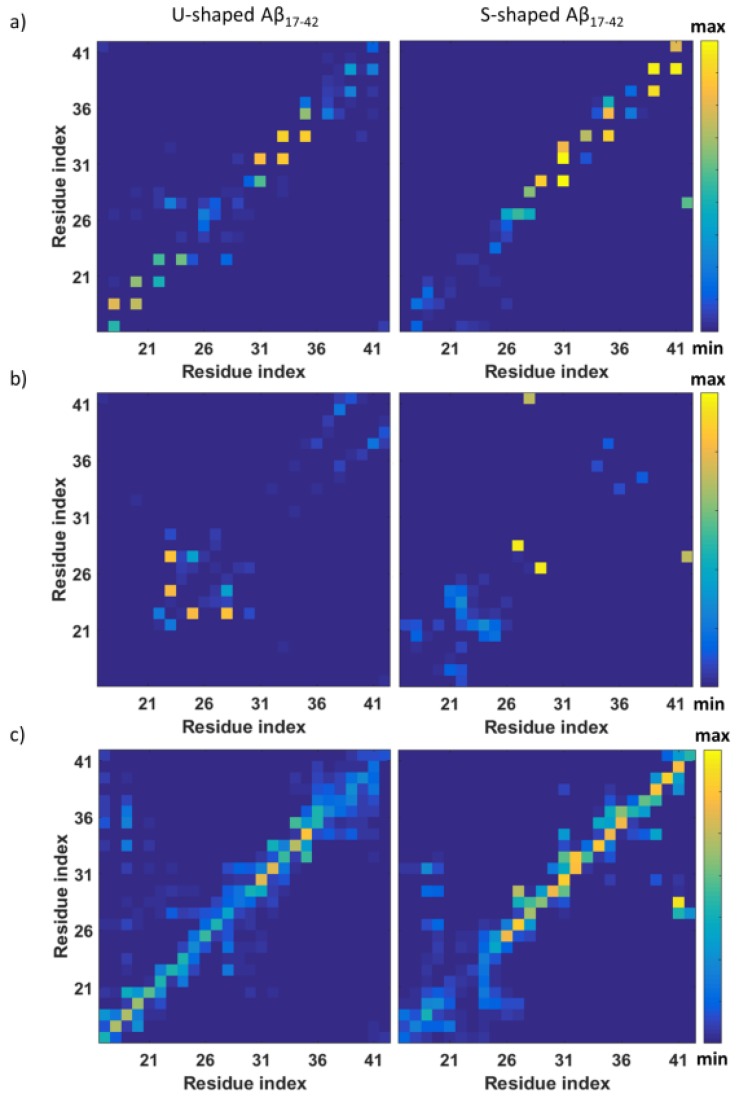
(**a**) U-shaped and S-shaped probability contact maps of inter-chain hydrogen bonds; (**b**) U-shaped and S-shaped probability contact maps of intra-chain hydrogen bonds; (**c**) U-shaped and S-shaped probability contact maps of inter-chain non-bonded contacts.

Figure 3a focuses on inter-chain hydrogen bonds, calculated using a cut-off of 0.35 nm [34]. The protein domain L17–D23 of the U-shaped model, in line with hydrophilic character of the buried surface, shows the highest probability of inter-chain hydrogen bonds (Figure 3a). Instead, the S-shaped arrangement showed a high probability contact in the central domain and C-terminal region (V39–A42). 

In addition to the inter-chain hydrogen bonds, we have also studied intra-chain H-bonds calculated within the same chain C using a cut-off of 0.35 nm. The highest probability of finding intra-chain H-bonds for the U-shaped model is between the side chain of residue D23 and backbone of G25 and sides chains of residues D23 and K28 (Figure 3b). The result is in agreement with previous literature indicating, in the U-shaped model, a salt bridge able to stabilize the loop region connecting two β-sheets preventing larger backbone motions [13,35,36].

It is worth mentioning that, in the central core, also the S-architecture presents two intra-chain contacts. The first one between the side chain of N27 and the backbone of G29, and a second one between side chains of A42 and K28 (Figure 3b), the latter identified earlier in the literature [17,23]. 

To get an overall view of inter-chain contacts, a map of all non-bonded interactions inside a cut-off =0.45 nm is shown in Figure 3c. The map clearly indicates how non-bonded interactions among same residues in an adjacent chain stabilize the S-shaped model particularly in the core and C-terminal region. In a greater detail, interactions between residues I41 and K28, G29 and A42 and K28 occurred only in the S-shaped model map. 

### 2.3. Order Parameter and Functional Mode Analysis 

**Figure 4:**
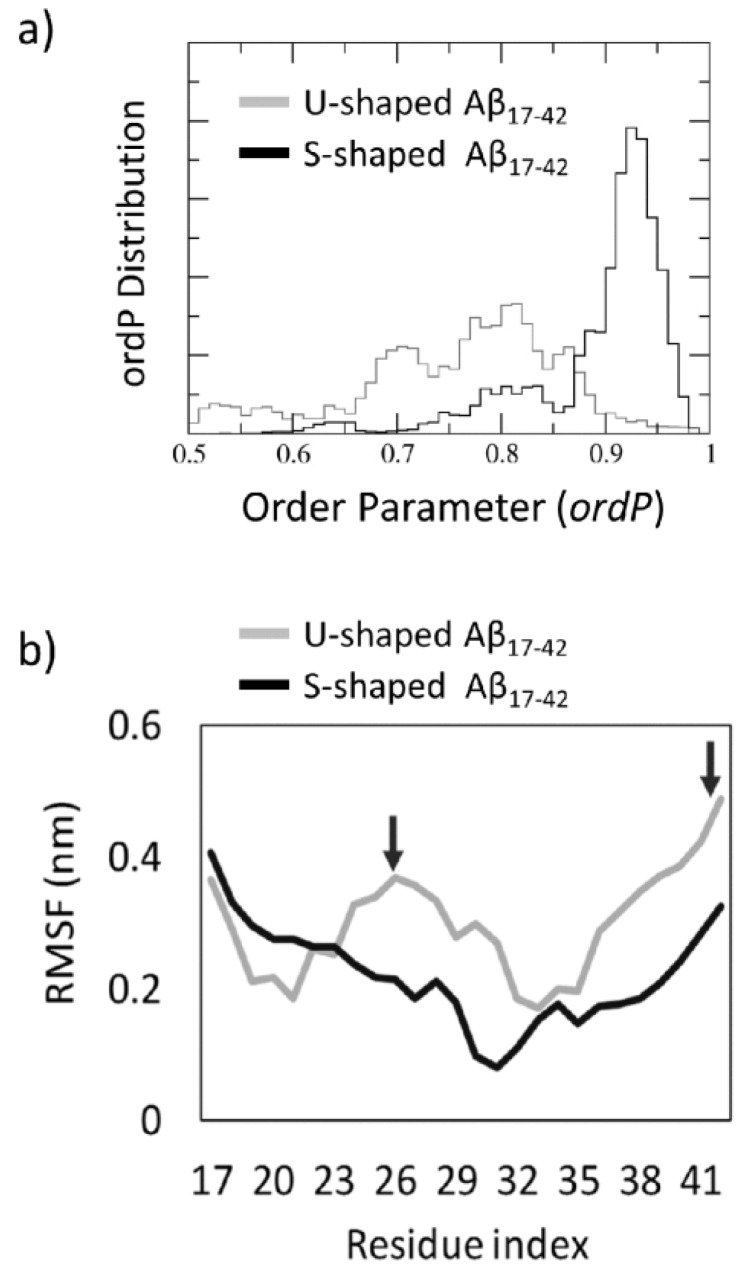
(**a**) Order Parameter, ordP, distribution calculated throughout the overall REMD trajectory at 300 K. The same number of snapshots was considered for both U-shaped (grey line) and S-shaped (black line) models. The ordP value provides a quantitative estimation of the fibril order. Values close to 1 indicate an alignment close to the starting structure, i.e., aligned fibre along the fibril axis. Values lower than 1 indicated a structure distortion; (**b**) Root Mean Squared Fluctuation (RMSF) plot calculated over the REMD trajectory at 300 K filtered on the ensemble weighted Maximally Correlated Motion (ewMCM) vector. Black arrows indicate residues with the highest RMSF in central and C-terminal regions of the U-shaped model.

The probability distribution of the order parameter, ordP (Supplementary Figure S3), calculated throughout the REMD trajectory at 300 K, is shown in Figure 4a. The S-shaped ordP along the overall 300 K REMD showed a sharp distribution with an average value and peak close to 0.95, thus indicating that the S-model maintains an intrinsic order of the fibre with chains aligned along the fibril axis. Instead, the U-shaped ordP has a spread distribution with a peak value around 0.8.

**Figure 5:**
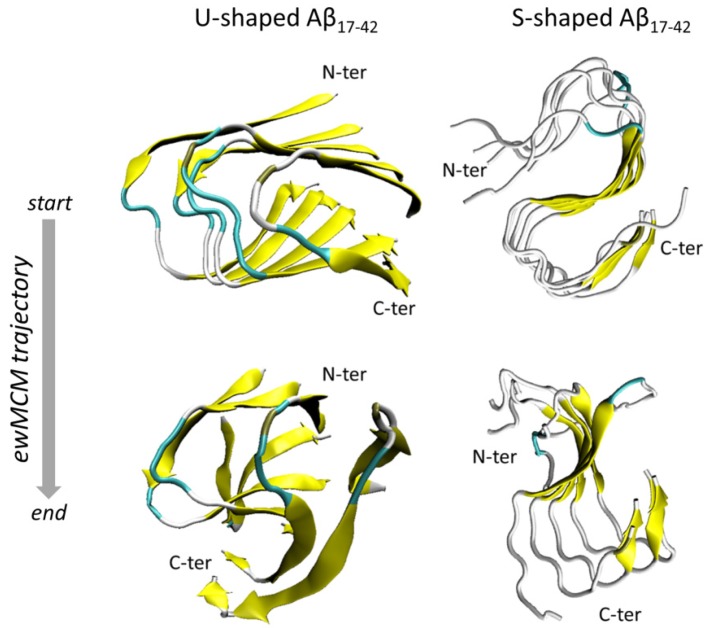
Snapshots representing the starting and the final configuration extracted from the ensemble weighted Maximally Correlated Motion (ewMCM) for U-shaped and S-shaped models after performing the Functional Mode Analysis (FMA) on the REMD ensemble at 300 K.

The Functional Mode Analysis (FMA) allowed to characterize the contribution of individual Principal Components Analysis (PCA) vectors to the fluctuation of ordP, yielding a single vector (the so-called ensemble weighted Maximally Correlated Motion, ewMCM), which drives the fibril structural destabilization (Supplementary Figures S4 and S5). Observing the residues RMSF (Figure 4b) calculated over the ewMCM trajectories (starting and final snapshots shown in Figure 5) a significantly different conformational behaviour can be observed for the U-shaped and the S-shaped models. 

Overall, the U-shaped model fluctuates much more than the S-shaped model, in particular for what concerns core and C-terminal regions (highlighted by arrows in Figure 4b). The C-terminal region is characterized by the highest fluctuation (RMSF_A42_ = 0.49 nm^2^) followed by the central loop area D23–I31 (RMSF_S26_ = 0.37 nm^2^). Instead, the S-shaped model, showed higher fluctuations of the N-terminal region, in agreement with inter-chain contact analysis (Figure 3) indicating a lack of inter-chain H-bonds in this region. 

In summary, the ordP shape factor and RMSFs calculated on ewMCM trajectories provided an interesting indication of the higher order maintained by the S-model under thermal motion, whereas the U-shaped model appeared to be more unstable and characterized by an overall disruptive conformational distortion (Figure 5). Furthermore, in agreement with previous data, provided by structural (Figure 1) and inter-chain analysis (Figure 2 and Figure 3), the S-shaped model seems to be subjected to a partial distortion only in the N-terminal region (L17–D23 region), whereas the U-shaped model assumed a more disordered configuration with a tendency to break in the central region losing almost completely the original conformation. 

## 3. Discussion

The major components of AD-associated amyloid plaques are Aβ_1–40_ peptides but also the more toxic Aβ_1–42_ species [7]. In the brain of patients affected by AD, those peptides build up, layer by layer, hierarchically organized assemblies. This molecular phenomenon is related to a progressive loss of brain function, especially memory loss and cognitive deficit, that becomes ultimately fatal. Amyloid fibrils exist in an equilibrium of interchanging structures of monomers and oligomers characterized by polymorphism [10,11,12,13,14,15]. 

Several structural models exist for the Aβ_1–40_ species, all sharing a U-shaped motif, made of two β strands (residues V18–S26 and residues I31–A40) linked by a central loop domain. In contrast, the Aβ_1–42_ species can also assume a S-shaped conformation [16,17,18,19,20,21], where three β strands (residues V12–V18, residues V24–G33, and residues V36–V40) are connected by major coil and turn region. It has been recently demonstrated that the S-shaped arrangement is not stable in case of Aβ_1–40_ [23]. Recently, the higher toxicity of Aβ_1–42_ species has been associated with its ability to assemble into ring-like N-fold models starting from the S-shaped fibril [22]. In this scenario, it is interesting to focus the attention on the two possible arrangements proposed for Aβ_1–42_ species. 

Results of the present research highlighted the S-shaped assembly as more stable when compared with the U-shaped model. Moreover, the U-shaped model showed a high degree of conformational plasticity, especially considering the high fluctuations of residues I41 and A42 (Figure 1). Our data are in apparent contradiction to literature published over the last decade classifying the U-shaped architecture as conformationally rather stable [37,38,39]. However, it is worth mentioning that the conformational sampling performed in previous computational studies was in general restricted to classical MD simulations and/or limited simulated time (from tens to hundreds of ns). In this view, our study should not be seen in contrast with previous literature, but as an improvement of the protein conformational sampling provided by REMD coupled with dimensionality reduction methods. 

Interestingly, the S-shaped model showed to maximize the formation of intra- and inter-chain hydrophobic contacts within the fibril model, especially on residues I41 and A42, characterized by a higher hydrophobic buried surface than the U-model. Nevertheless, the most significant contribution to the stability of S-model is attributed to the inter-chain hydrophobic (Figure 2c) and hydrophilic (Figure 2d) interaction surface, especially in the C-terminal region. More in depth, in agreement with a recent computational study [23], our results showed the following inter-chain hydrophobic contacts only in the S-shaped model: I41–K28, I41–G29, and A42–K28. The fundamental role played by those residues was also confirmed by analysing the inter-chain contacts (Figure 2a). Even the FMA analysis highlighted the importance of the C-terminal region, which showed to be much more stable, than the N-terminal one. In contrast, the U-shaped model was affected by a higher distortion, which started from the core region related to inter-chain contacts disruption. The above-mentioned observations (Figure 5) were quantified by RMSFs profiles (Figure 4b).

It is important to remark, that, for the sake of a meaningful comparative analysis, the same protein region (residues L17–A42) has been considered for both U-shaped and S-shaped models. The neglected domain is known to be unstructured and not present in the U-shaped PDB file (2BEG [12]). On the other hand, the S-shaped PDB model (2MXU [17]) contains an additional structured region between residues E11–K16. It is reasonable to consider that neglecting the above mentioned region may somehow affect the whole S-shaped arrangement toward a higher or a lower stability. In this connection, Figure 2a and Figure 4b provide a first indication on possible effects on the overall protein assembly stability. In a greater detail, whereas the U-architecture instability is related with the central area and C-terminals regions, the N-terminal tail (residues L17–V24) represents the weakest area of the S-architecture. The presence of the E11–K16 structured domain is reasonably expected to strengthen and further stabilize the inter-chain hydrogen bonds of protein region L17–V24. A convincing demonstration of the above mentioned hypothesis is shown in Supplementary Figure S6. As expected, the S-shaped_11–42_ model, showed a higher intrinsic order with respect to the S-shaped_17–42_ (Figure S6). 

Summarizing, the existence of U-shaped and S-shaped assemblies for the Aβ_1–42_ species has been already demonstrated by several previous studies [12,16,17,18,19,20,21] and not under discussion in the present work. Instead, outcomes of the present comparative study, provided clear information on the tendency of a specific conformational state to explore and eventually get out of the free energy minimum identified by the correspondent experimental model. Our data, based on 6 μs of enhanced conformational sampling for each model, clearly suggest the U-shaped is much less stable than S-shaped model, at least for what concerns a Aβ_17–42_ pentamer. 

Nonetheless, previous computational studies have focused on U-shaped models to investigate the ligand driven destabilization of Aβ_1–42_ species [39,40,41,42]. In this regard, our data suggest that enhanced sampling techniques may be a valuable and powerful tool to shed light on the relationship between ligand-protein interactions and protein structural modifications. Moreover, in the specific case of Aβ_1–42_ species, the S-shaped model should also be considered as a target for rational design/discovery/optimization of effective compounds. 

## 4. Material and Methods

### 4.1. Replica Exchange Molecular Dynamics (REMD)

Two different models for the Aβ_1–42_ species were considered: the U-shaped Aβ_17–42_ (PDB ID: 2BEG [12]) and the recently resolved S-shaped Aβ_11–42_ (PDB ID: 2MXU [17]). Starting models are also reported in Supplementary Figure S1. A pentamer of Aβ_17–42_ was extracted from each PDB structure. In the manuscript the considered pentamers are called, the U-shaped model (Aβ_17–42_ pentamer extracted from 2BEG.pdb [12]) and the S-shaped model (Aβ_17–42_ pentamer extracted from 2MXU.pdb [17]).

The U-shaped and the S-shaped models were solvated in a cubic box with each side equal to 6 nm and neutralized by counterions. Each system consisted of about 21,000 interacting particles. 

The AMBER99SB-ILDN force field [43] was used to define protein topologies and the TIP3P model [44] was used to represent the water molecules. The systems were first minimized by applying the steepest descent energy minimization algorithm, followed by preliminary simulation in NVT ensemble (constant Number of particles, Volume, and Temperature) of 50 ps duration. V-rescale thermostat was applied to keep temperature at 300 K with a time constant of 0.1 ps [45]. An additional simulation in NPT ensemble (constant Number of particles, Pressure, and Temperature) of 50 ps duration was carried out at 300 K (τ = 1 ps) and 1 atm (τ = 5 ps). V-rescale [45] and Berendsen [46] coupling methods were used as temperature and pressure coupling. Then, 100 replicas were generated with temperatures ranging from 280 to 558 K and distributed applying the exponential spacing strategy, as previously done in literature [47,48]. A first NVT position restrained MD was run on each replica for 50 ps. Finally, a 60 ns of production NVT-REMD was carried out on each replica at its own temperature, according to previous works [49]. The replica exchange interval was set equal to 1 ps, large enough if compared to the time constant of the heath bath (τ = 0.1 ps). The resulting exchange probability was 0.3. The computational data were time-averaged over all trajectory steps corresponding to the chosen temperature, 300 K in this work. The LINCS algorithm [50] was used to constrain the lengths of all bonds. The integration time step was 2 fs. Periodic boundary conditions were applied along *xyz*. The short-range Van der Waals (VDW) and electrostatics interactions were cut off after 1 nm; the Particle Mesh Ewald (PME) method [51] was employed for long-range electrostatics. GROMACS 5 was used for REMD simulations and data analysis [52]. The inter-chain protein contacts were identified by contact probability plots. Contact probability for each residue was calculated as already described in a previous work [49].

### 4.2. Order Parameter and Functional Mode Analysis (FMA)

With the purpose of estimating the structural order of the two models and therefore how much protein chains are aligned, an order parameter was calculated for each REMD snapshot as follows:

(1) $$ordP=\frac{1}{N_{r}}\overset{42}{\underset{r=17}{\sum }}\frac{\langle \nu_{r},\;z\rangle }{\left\|\nu_{r}\|\cdot \|z\right\|}=\frac{1}{N_{r}}\overset{42}{\underset{r=17}{\sum }}\cos\alpha$$

In Equation (1), *v_r_* is the vector joining each of the *N_r_* C_α_-atoms pertaining to chain A with the corresponding C_α_-atom (same residue number) of chain E and *z* is the fibril axis. Values of ordP close to 1 indicated an alignment close to the initial structure, i.e., aligned fibre along the fibril axis *z*. Values of ordP lower than 1 indicated a structure distortion (also refer to Supplementary Figure S3). 

Functional mode analysis (FMA) was applied to the REMD trajectory at 300 K [53] to elucidate collective motions directly related to fibre distortion. The applied method detects a collective motion maximally correlated to the fluctuation of the quantity of interest, that is, in the case under study, the above mentioned order parameter. Assuming that the variable of interest is a linear function of the Principal Components, the maximally correlated vector can be derived by maximizing the Pearson coefficient [53] to quantify the contributions of the individual PCA vectors to the fluctuations of the variables of interest. This approach yields a single collective mode, which drives the phenomenon under investigation, referred to as ensemble-weighted Maximally Correlated Motion (ewMCM). In applying FMA, it is crucial to cross-validate the derived model for an independent set of simulation frames. The established approach applied for cross-validating the obtained results is to divide the simulation into a subset of frames for model building and a subset of frames for cross-validation. In this work, the obtained maximally correlated motion was validated by predicting the function of interest, in the cross-validation subset, with Pearson correlation coefficient higher than 0.93 for U-shaped and 0.97 for S-shaped models. Further details of the FMA calculation are provided as Supplementary Figures S4 and S5.

## Supplementary Materials

Supplementary materials can be found at http://www.mdpi.com/1422-0067/19/2/571/s1.
