# Supplementary material for: Conformational Dynamics and Stability of U-Shaped and S-Shaped Amyloid β Assemblies
Source: Int J Mol Sci. 2018 Feb 14;19(2):571. doi: 10.3390/ijms19020571 (PMC5855793; doi:10.3390/ijms19020571)
Supplement: Supplementary file 1 [file ijms-19-00571-s001.pdf]

SUPPORTING INFORMATION

S1.1 Investigated Models

The U-shaped model: a pentamer of Aβ_{17-42} extracted from 2BEG.pdb file (Figure S1A).

The S-shaped model: a pentamer of Aβ_{17-42} extracted from 2MXU.pdb file (Figure S1B).

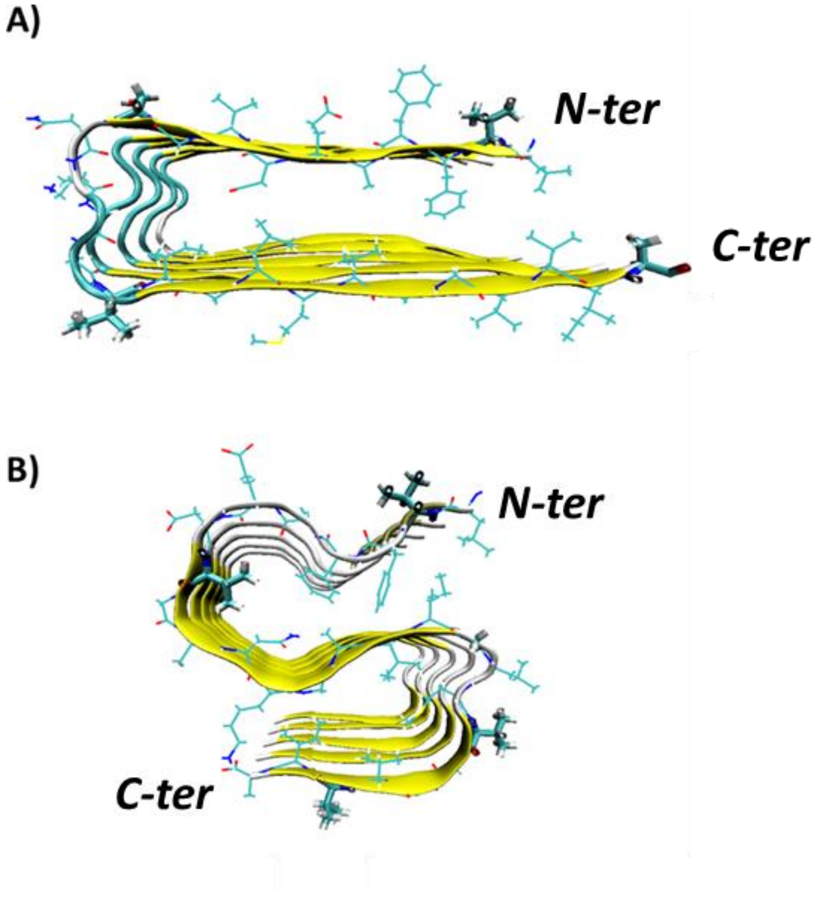

Figure S1: A) U-shaped and B) S-shaped models

S1.2 Conformational Analysis of the REMD Ensemble at 300K

The Solvent Accessible Surface Area (SASA) together with the estimation of its hydrophobic and hydrophilic component is shown in (Figure S2A). The total SASA of U-shaped model (76.07 ± 4.17 nm^2) is slightly higher than that of the S-shaped model (70.62 ± 3.71 nm^2). This result may be related to the S-shaped model ability to achieve a slightly more compact arrangement, as also shown by the Radius of Gyration (Figure S2B).

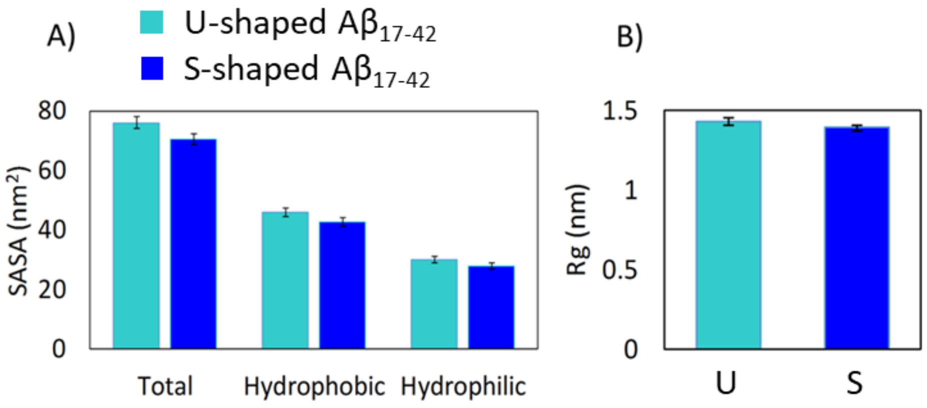

Figure S2: A) Total, Hydrophobic and Hydrophilic SASA of the two models compared. B) Radius of gyration of the two models.

S1.3 Definition of the Order Parameter

With the purpose of comparing the structural order of the U-shaped and S-shaped models, an order parameter was defined.

$$ordP = \frac{1}{N_r}\sum_{r=17}^{42}\frac{\langle v_r, z\rangle}{\|v_r\|\cdot\|z\|} = \frac{1}{N_r}\sum_{r=17}^{42}\cos\alpha \qquad \text{eq.S1}$$

In *eq.S1*, v_r is the vector joining the N_r C_α-atoms pertaining to chain A with the corresponding C_α-atoms of chain E (i.e., on the same residue number) and z is the fibril axis. Values of *ordP* close to 1 indicate the amyloid-like shape alignment, whereas values of *ordP* much lower than 1 are typical of a distorted structure (Figure S3).

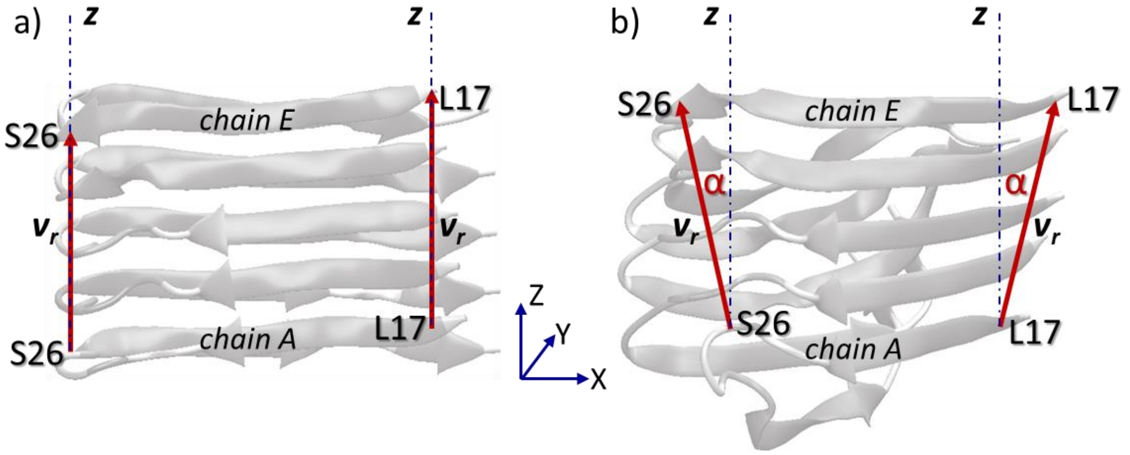

Figure S3: Schematic representation of the main players in determining the ordP value as defined in eq.S1. The ordP value is equal to 1 in a completely aligned fiber (a), and lower than 1 for a distorted fiber (b).

S1.4 Functional mode analysis

In Figure S4 the scatter plot of the data-model is reported for the two assemblies. The Pearson correlation coefficient is high for both models.

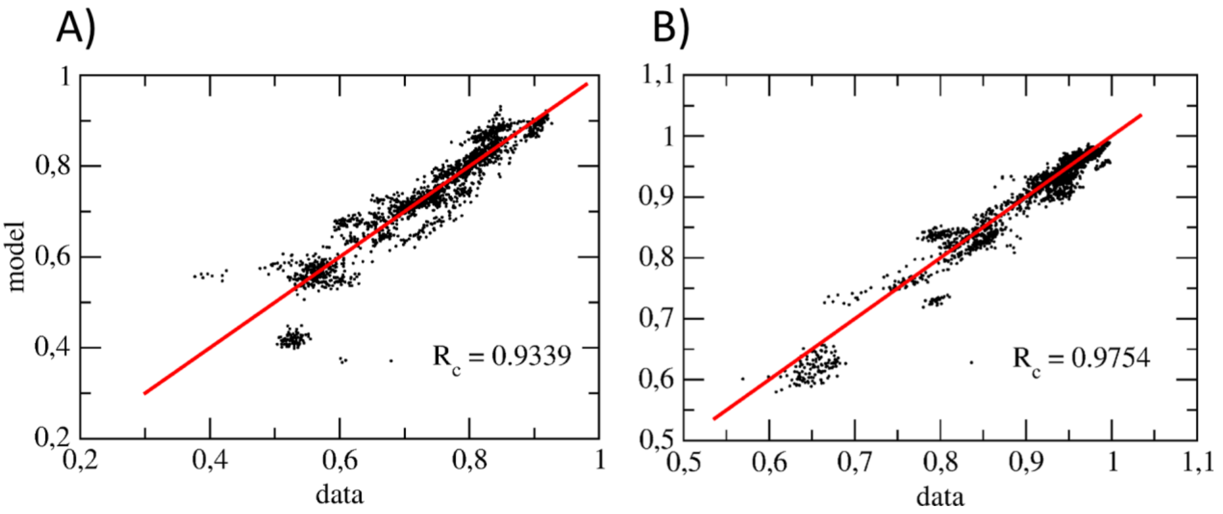

Figure S4. Scatter plots of the data-model for A) U-shaped and B) S-shaped model. In red the line corresponding to the cross-validation is reported.

The cross-validation of the maximally correlated motion was made by dividing the simulation in two parts: one for model building and the second for cross-validation as shown in Figure S5:

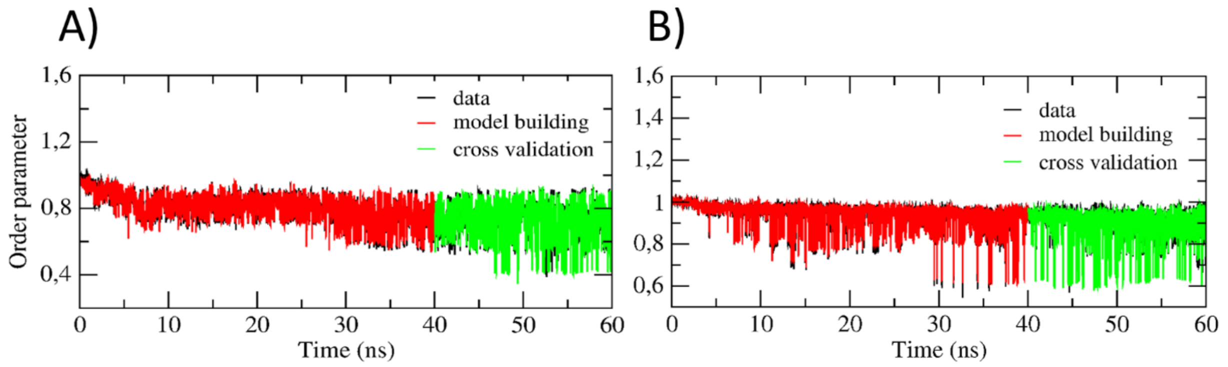

Figure S5. Order parameter as a function of time (black) obtained from REMD trajectories of U-shaped (A) and S-shaped (B) model. The model building curve (red) and the cross validation curve (green) are reported.

S1.5 Stability of the S-shaped Aβ17-42 compared to Aβ11-42

A pentamer of $A\beta_{11-42}$ extracted from 2MXU.pdb file was considered. As done for the $A\beta_{17-42}$ U-shaped and S-shaped models, the system was solvated by adding explicitly modelled water and ions. The simulation set-up in terms of system minimization, classical and replica exchange MD follows the same procedure described in the main text for the $A\beta_{17-42}$ U-shaped and S-shaped models. The ensemble at 300K resulting from the REMD on the S-shaped $A\beta_{11-42}$ was considered to quantify (*eq. S1*) the order parameter, *ordP*. The *ordP* distribution was then compared with the one estimated on the conformational ensemble at 300K obtained by REMD simulations carried out on the S-shaped $A\beta_{17-42}$ model (Figure S6).

Results clearly highlight the stabilizing role played by residues E11-K16.

Hence, the comparison between the $A\beta_{17-42}$ U-shaped and S-shaped models presented in the main manuscript can be reasonable considered as not affected by the choice to neglect the residue range E11-K16 in the 2MXU.pdb file. On the other hand, that choice made meaningful the comparative analysis between the U-shaped and S-shaped models.

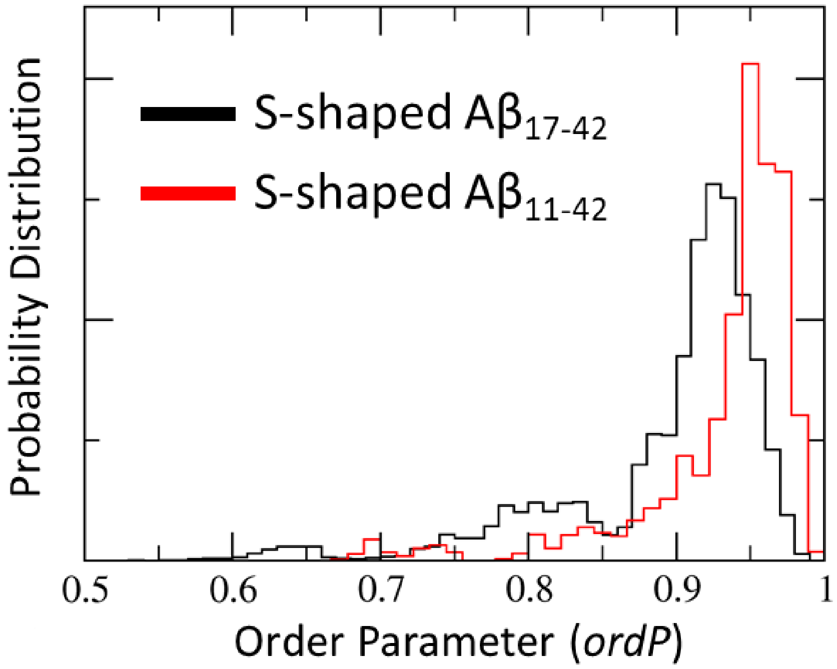

Figure S6. a) *ordP* distribution of the $A\beta_{17-42}$ S-shaped model and the $A\beta_{11-42}$ S-shaped model. The ordP distribution has been calculated throughout the overall REMD trajectory at 300K taking the same number of snapshots for both models. It is worth mentioning how the structured protein portion E11-K16 provides support toward a higher fibril order.
